# 

**Published:** 1903-10-17

**Authors:** 


### CONTENTS.

**King's College Hospital** - 41

**Ankylostomiasis in Cornwall** - 42

**Annotations** - 43

**Notes and News** - 44

**Hospital Clinics and Medical Progress—**

Cardiac Aneurysms - 45

Carbolic Acid and the Treatment of Plague - 45

Operation in Carcinoma of the Breast - 46

Typhoid Fever in Infancy and Childhood - 47

Progress in Phototherapy and Electro-therapeutics - 48

Progress in Nerve Surgery - 49

Progress in Surgery of the Liver and Gall Bladder - 50

**New Appliances and Things Medical** - 51

**Hachish in the Old Testament** - 52

**Hospital Administration—**

King's College Hospital - 53

Glances at the Hospitals - 57

**Editor's Letter-Box** - 57

**The Student of Medicine** - 58

*NURSING SECTION.*

**Notes on News from the Nursing World—**

Our Christmas Distribution—Queen Alexandra's Military Nursing Service—Mr. Alfred Lyttelton and Colonial Nursing—Victorian Trained Nurses' Association—The New Bishop of Manchester and Nursing—The Nurses of Mount Vernon Hospital—Superfluous Nurses at the Asylums Board Hospitals—One Nurse to Forty Patients—Pauper Nursing Difficulties—A New Institute for the Training of Nurses—Opening of the Memorial Home at Ripon—Darlington Queen's Nurses' Association—Nurses and Bicycles—Successful Probationers at Bradford Infirmary—The Testimonial Question—The Granard Scandal—Belfast "Nurses' Own"—Short Items - 38-40

**Lectures on Ophthalmic Nursing** - 41

**To Nurses** - 41

**Irish Dispensary Midwives and their Work** - 42

**First Night in a Private Nurses' Home** - 42

**Hints on How to Start a District Nursing Association** - 43

**Gynæcological Nursing** - 44

**Nursing in the Netherlands** - 45

**"The Hospital" Convalescent Fund** - 45

**Everybody's Opinion** - 46

**Appointments** - 47

**Presentations** - 47

**The Nurses' Bookshelf** - 47

**Echoes from the Outside World** - 48

**A Book and its Story** - 49

**For Reading to the Sick** - 50

**Notes and Queries** - 50

